# Supplementary material for: Tolerance of Honey Bees to Varroa Mite in the Absence of Deformed Wing Virus
Source: Viruses. 2020 May 23;12(5):575. doi: 10.3390/v12050575 (PMC7290856; doi:10.3390/v12050575)
Supplement: Supplementary file 1 [file viruses-12-00575-s001.pdf]

viruses

Tolerance of honey bees to *Varroa* mite in the absence of deformed wing virus

John MK Roberts, Nelson Simbiken, Chris Dale, Joel Armstrong and Denis L Anderson

Table S1. Collection information of honey bee and Varroa mite samples used in this study.

Date	Latitude	Longitude	Location	Province	Country	Species	RNAseq	RT–qPCR
May 2008	−6.084	145.386	Goroka	EHP	PNG	*V. jacobsoni*	yes	no
May 2008	−6.084	145.386	Goroka	EHP	PNG	*V. jacobsoni*	yes	no
May 2014	−5.935	143.279	Malke	Hela	PNG	*A. mellifera*	yes	yes
May 2014	−5.827	144.407	Minj	Jiwaka	PNG	*A. cerana*	no	yes
May 2014	−5.827	144.407	Minj	Jiwaka	PNG	*A. mellifera*	yes	yes
May 2014	−5.811	144.589	Banz	Jiwaka	PNG	*A. mellifera*	yes	yes
May 2014	−6.019	144.910	Donakanage	Chimbu	PNG	*A. mellifera*	yes	yes
May 2014	−6.035	144.960	Mirane	Chimbu	PNG	*A. mellifera*	yes	yes
May 2014	−5.833	145.099	Kegesuglo	Chimbu	PNG	*A. mellifera*	yes	yes
May 2014	−6.100	145.133	Mangiro	Chimbu	PNG	*A. mellifera*	yes	yes
May 2014	−6.050	145.020	Ku	Chimbu	PNG	*A. mellifera*	yes	yes
May 2014	−5.991	145.378	Kabiufa	EHP	PNG	*A. mellifera*	yes	yes
May 2014	−6.073	145.395	Bena	EHP	PNG	*A. mellifera*	yes	yes
May 2014	−6.145	145.402	Kamaliky	EHP	PNG	*A. mellifera*	yes	yes
May 2014	−5.973	145.475	Komunive	EHP	PNG	*A. mellifera*	yes	yes
May 2014	−6.129	145.562	Amayufa	EHP	PNG	*A. mellifera*	yes	yes
May 2014	−6.429	145.674	Irafo	EHP	PNG	*A. mellifera*	yes	yes
Sep 2014	−10.457	161.918	Freshwinds	Makira	Solomon Islands	*A. cerana*	no	yes
Sep 2014	−10.436	161.802	Waimapuru	Makira	Solomon Islands	*A. cerana*	no	yes
Sep 2014	−10.411	161.758	Tawapuna	Makira	Solomon Islands	*A. cerana*	no	yes
Sep 2014	−10.463	161.867	Manchuki	Makira	Solomon Islands	*A. cerana*	no	yes
Sep 2014	−8.767	160.695	Auki	Malaita	Solomon Islands	*A. mellifera*	yes	yes

Sep 2014	−8.733	160.703	Aimela	Malaita	Solomon Islands	*A. mellifera*	yes	yes
Sep 2014	−8.704	160.683	Gwaunaru'u	Malaita	Solomon Islands	*A. mellifera*	yes	yes
Sep 2014	−8.590	160.685	Dala	Malaita	Solomon Islands	*A. mellifera*	yes	yes
Sep 2014	−8.767	160.663	Fiu	Malaita	Solomon Islands	*A. mellifera*	yes	yes
Sep 2014	−8.917	160.768	Arabala	Malaita	Solomon Islands	*A. mellifera*	yes	yes
Sep 2014	−8.917	160.768	Arabala	Malaita	Solomon Islands	*A. mellifera*	yes	yes
Sep 2014	−8.767	160.695	Betlem	Malaita	Solomon Islands	*A. mellifera*	yes	yes
Sep 2014	−8.959	160.772	Betlem	Malaita	Solomon Islands	*A. mellifera*	yes	yes
Nov 2015	−6.009	145.313	Asaro	EHP	PNG	*A. mellifera*	no	yes
Nov 2015	−5.962	145.260	Anegu	EHP	PNG	*A. mellifera*	no	yes
Nov 2015	−6.101	145.076	Ku	Chimbu	PNG	*A. mellifera*	no	yes
Nov 2015	−6.012	144.966	Kundiawa	Chimbu	PNG	*A. mellifera*	no	yes
Nov 2015	−6.035	144.960	Windy Lodge	Chimbu	PNG	*A. mellifera*	no	yes
Nov 2015	−5.922	144.849	Kenowagi	Chimbu	PNG	*A. mellifera*	no	yes
Nov 2015	−5.858	144.667	Minj	Jiwaka	PNG	*A. mellifera*	no	yes
Nov 2015	−5.793	144.627	Banz	Jiwaka	PNG	*A. mellifera*	no	yes
Nov 2015	−5.678	144.399	Penga	Western Highlands	PNG	*A. mellifera*	no	yes
Nov 2015	−5.860	144.219	Mt Hagen	Western Highlands	PNG	*A. mellifera*	no	yes
Nov 2015	−5.515	144.149	Baiyer Valley	Western Highlands	PNG	*A. mellifera*	no	yes
Nov 2015	−6.336	145.885	Aiyura	EHP	PNG	*A. cerana*	no	yes
Nov 2015	−6.012	145.325	Asaro	EHP	PNG	*A. cerana*	no	yes
Nov 2015	−5.858	144.667	Minj	Jiwaka	PNG	*A. cerana*	no	yes
Nov 2015	−5.793	144.627	Banz	Jiwaka	PNG	*A. cerana*	no	yes
Nov 2015	−5.835	144.509	Kudjip	Jiwaka	PNG	*A. cerana*	no	yes
Nov 2015	−5.516	144.150	Baiyer Valley	Western Highlands	PNG	*A. cerana*	no	yes
May 2018	−6.062	145.399	Goroka	EHP	PNG	*A. cerana*	yes	yes
May 2018	−6.157	145.365	Oiafaiyufa	EHP	PNG	*A. mellifera*	yes	yes
May 2018	−6.254	145.435	Kemasi	EHP	PNG	*A. mellifera*	yes	yes
May 2018	−6.248	145.434	Kemasi	EHP	PNG	*A. mellifera*	yes	yes

May 2018	−6.101	145.389	Goroka	EHP	PNG	*A. mellifera*	yes	yes
Oct 2018	−6.254	145.435	Kemasi	EHP	PNG	*A. mellifera*	yes	yes
Oct 2018	−6.248	145.434	Kemasi	EHP	PNG	*A. mellifera*	yes	yes
Oct 2018	−6.184	145.425	Bena	EHP	PNG	*A. mellifera*	yes	yes
Oct 2018	−6.019	145.291	Asaro	EHP	PNG	*A. mellifera*	yes	yes
Oct 2018	−6.062	145.399	Goroka	EHP	PNG	*A. cerana*	yes	yes
Oct 2018	−6.062	145.399	Goroka	EHP	PNG	*A. mellifera*	yes	yes
Oct 2018	−6.062	145.399	Goroka	EHP	PNG	*A. cerana*	yes	yes
Oct 2018	−6.128	145.429	Bena	EHP	PNG	*A. mellifera*	yes	yes
Oct 2018	−6.086	145.397	Goroka	EHP	PNG	*A. mellifera*	yes	yes
Oct 2018	−6.020	144.970	Mirane	Chimbu	PNG	*A. mellifera*	yes	yes
Oct 2018	−6.020	144.970	Mirane	Chimbu	PNG	*A. mellifera*	yes	yes
Oct 2018	−6.050	145.020	Ku	Chimbu	PNG	*A. mellifera*	yes	yes
Oct 2018	−6.118	145.131	Chuave	Chimbu	PNG	*A. cerana*	yes	yes
